# High‐Dependency Care Without Mechanical Ventilation for Severe Guillain–Barré Syndrome in a Rural Low‐Income Setting

**DOI:** 10.1155/crcc/1700879

**Published:** 2026-05-12

**Authors:** Lesley Notghi, Louise Little, Joel Chin

**Affiliations:** ^1^ Department of Medicine, Hopitaly Vaovao Mahafaly, Mandritsara, Madagascar; ^2^ High-Dependency Unit, Hopitaly Vaovao Mahafaly, Mandritsara, Madagascar; ^3^ Department of Anaesthesia, Cambridge University Hospitals NHS Foundation Trust, Cambridge, UK, cuh.org.uk

## Abstract

Guillain–Barré syndrome (GBS) is a major cause of neuromuscular respiratory failure globally. In many low‐income settings, access to mechanical ventilation and disease‐modifying therapy is limited. We describe the management of a young woman with rapidly progressive GBS and bulbar weakness in a mission hospital in northern Madagascar without access to intensive care facilities, invasive ventilation or immunotherapy. Respiratory failure was managed using a structured high‐dependencyunit (HDU) framework combining noninvasive respiratory support with intensive nursing care, physiotherapy, regular repositioning and close nurse‐led observation. The patient survived the acute phase, regained swallow and speech by Day 8 and was discharged from HDU on Day 15. At 1‐year follow‐up, she had recovered full upper limb strength with residual lower limb weakness. This case highlights the potential for structured supportive care in an HDU to sustain life in clinically significant neuromuscular respiratory failure when definitive critical care resources are unavailable.

## 1. Introduction

Guillain–Barré syndrome (GBS) is the leading cause of acute flaccid paralysis worldwide, and up to 20% of patients require ventilatory support [1]. Respiratory compromise in GBS reflects a combination of neuromuscular weakness, bulbar dysfunction and impaired airway clearance. In high‐income settings, outcomes have improved through access to mechanical ventilation and immunotherapy, including intravenous immunoglobulin (IVIG) and plasma exchange.

In contrast, in low‐income countries, most district hospitals lack access to intensive care facilities, invasive ventilation and disease‐modifying therapy, turning a potentially reversible neurological emergency into a life‐threatening condition. In such settings, management depends on meticulous supportive care encompassing respiratory support, airway protection, nutrition, thromboprophylaxis, skin care, physiotherapy and prevention of secondary complications. We describe the management of a young woman with rapidly progressive paralysis and bulbar weakness in a mission hospital in northern Madagascar with no access to intensive care facilities. This case highlights how structured care delivered in a high‐dependency unit (HDU) can sustain life in severe neuromuscular respiratory failure when invasive ventilation and immunotherapy are unavailable.

## 2. Case Presentation

A previously healthy 24‐year‐old woman had a self‐terminating attack of fever and diarrhoea, followed several days later by ascending weakness, urinary retention and dysarthria. On admission, she was alert but tachypnoeic (≈60 min^−1^) with shallow, diaphragmatic breathing but oxygen saturation maintained on room air. Power was 0/5 in the lower limbs and 1/5 in the upper limbs, with areflexia and preserved sensation. Cranial‐nerve testing revealed dysarthria and dysphagia but normal ocular and facial movements. Muscle weakness reached its nadir within approximately 1 week, consistent with the typical time course of acute inflammatory demyelinating polyneuropathy [2]. Muscle strength, assessed using the Medical Research Council (MRC) sum score (range 0–60) based on bilateral testing of six muscle groups, was 4/60.

Routine investigations were unremarkable. Schistosomiasis serology and HIV testing were negative. Cerebrospinal‐fluid protein measurement and nerve conduction studies were unavailable. The rapid, symmetrical, ascending paralysis with areflexia and preserved sensation was most consistent with a clinical diagnosis of GBS, but neither IVIG nor plasma exchange—the standard therapies—was accessible.

The differential diagnoses included neurological causes such as transverse myelitis, botulism, brainstem stroke and neuromuscular junction disorders including myasthenia gravis, as well as acute inflammatory myopathies. However, the rapid progression, generalised areflexia, absence of a sensory level and subsequent recovery pattern made these less likely.

Given the rapid disease progression, bulbar involvement and MRC sum score, the Erasmus GBS Respiratory Insufficiency Score (EGRIS) was 7, the maximum score, corresponding to a high risk of ventilatory failure [3]. The patient was transferred to the newly established surgical HDU. She was unable to swallow or cough, and frequent secretion pooling caused desaturations down to approximately 80%, requiring urgent medical review and intervention. Intubation and prolonged ventilation were not feasible because our hospital possessed only two home ventilators and limited sedative agents (ketamine, diazepam, small quantities of propofol), making long‐term ventilation unreliable and unsafe.

Staffing consisted of two nurses per shift, during the day and overnight, covering all monitored beds. Depending on occupancy, the nurse‐to‐patient ratio ranged from 1:2 to 1:4. The nurses had ward‐based experience and had undergone a month‐long structured training programme based on an ABC approach to acute illness, including early recognition of deterioration, use of a modified early warning score (MEWS), escalation protocols and principles of frequent monitoring and basic critical care support.

In this context, we implemented a structured noninvasive respiratory‐support bundle delivered by nurses. Intermittent noninvasive ventilation (NIV) provided positive‐pressure assistance (IPAP 11 cm H2O, EPAP 5 cm H2O) as tolerated. Between sessions, breath‐stacking and lung‐volume‐recruitment manoeuvres were performed using a Mapleson C circuit with a one‐way valve to maintain alveolar inflation and augment cough effort [4].

Care was delivered using a protocolised nursing observation chart mandating 2‐hourly documentation across a 24‐h cycle, including vital signs, airway surveillance, fluid balance, catheter checks and pressure‐area prevention. Repositioning, suctioning and airway clearance were therefore structured rather than ad hoc. Close family members were present throughout admission and assisted with basic nonclinical supportive care, including comfort measures and repositioning, under nursing supervision.

The HDU respiratory support bundle comprised the following:

• Regular chest physiotherapy with manual percussion and assisted coughing,
• Regular repositioning using postural‐drainage positions,
• Regular suctioning of oral secretions,
• Gradual enteral feeding via nasogastric tube and bowel management,
• Thromboprophylaxis with a limited supply of direct oral anticoagulant donated by a visiting volunteer and used under hospital supervision.

By Day 8, she had regained cough and speech. Swallow assessment using a bedside water test confirmed safe swallowing, and oral intake was gradually resumed. Over the subsequent week, the MRC sum score improved to 21/60, in parallel with recovery of cough, speech and swallowing. During recovery, simple bedside measures including peak expiratory flow were used to follow cough strength, increasing from approximately 80 to 200 L·min^−1^ prior to discharge. By Day 13, she had progressed to self‐feeding and was discharged from HDU on Day 15. At 1‐year follow‐up, she had recovered full upper limb strength, but lower limb weakness persisted.

## 3. Discussion

This case illustrates that, in patients with GBS‐related respiratory failure requiring close monitoring, careful supportive management may sustain life in the absence of invasive ventilation or immunotherapy. In many low‐income hospitals, IVIG and plasma exchange are infrequently used [5, 6]; and mechanical ventilation is often unavailable[6, 7].

Mortality from GBS remains higher in resource‐limited settings than in high‐income environments. In a recent two‐centre cohort study in Ethiopia, 65.8% of patients admitted to ICU required mechanical ventilation, with an overall ICU mortality of 19.2%; only 15% of patients received IVIG [6]. Aspiration and cardiac arrest were common complications, reflecting the central role of respiratory failure in determining outcome. The authors attributed higher mortality compared with high‐income settings to disparities in access to and quality of intensive care. In many low‐resource hospitals, however, the capacity to deliver prolonged invasive ventilation safely is limited, making alternative strategies for respiratory support essential.

In our institution, a small home ventilator had previously been used for selected surgical patients with poor outcomes due to limited sedative choice and inability to manage prolonged ventilation. Against this backdrop, our structured HDU model represents a pragmatic approach to delivering supportive care when definitive critical care resources are unavailable.

The combination of intermittent NIV, Mapleson C breath‐stacking, physiotherapy and meticulous nursing care achieved the key physiological goals of invasive ventilation—maintaining alveolar recruitment, secretion clearance and gas exchange—using only simple equipment. Nurse‐led vigilance was central: continuous observation enabled early detection of respiratory distress and prompt suctioning, while regimented repositioning and postural drainage reduced atelectasis risk.

The HDU operated with two nurses per shift for up to eight monitored beds. In this constrained staffing environment, the protocolised 2‐hourly observation chart functioned as a surrogate for formal ICU systems, embedding discipline and consistency into care delivery despite limited technology.

Our approach aligns with calls to develop models of critical care that bridge ward and ICU care in resource‐limited settings [7]. Effective HDUs rely on skilled nursing, monitoring and disciplined routines rather than complex infrastructure. The successful outcome of this case demonstrates that organised teamwork and training can offset equipment scarcity.

While diagnostic confirmation and disease‐specific therapy were unavailable, the clinical course and recovery pattern were typical of GBS. The absence of IVIG and plasma exchange may have contributed to the slower lower limb recovery observed at 1‐year follow‐up, limiting generalisability. Recovery in GBS typically unfolds over weeks to months and may continue throughout the first year after onset [1]. Recovery timelines in low‐ and middle‐income settings remain under‐reported in the literature, making direct comparison difficult.

This case nevertheless demonstrates that structured, protocolised supportive care can sustain life through the period of greatest respiratory vulnerability in severe GBS. In settings where invasive ventilation and immunotherapy are unavailable, disciplined nursing care, close monitoring and simple respiratory adjuncts may bridge the acute phase of neuromuscular respiratory failure. The interventions described here are reproducible, low cost and require minimal technology—an adaptable model for similar hospitals across sub‐Saharan Africa.

## Author Contributions

Lesley Notghi: clinical management, conceptualisation, manuscript revision.

Louise Little: nursing care coordination, data collection, manuscript revision.

Joel Chin: clinical management, conceptualisation, manuscript drafting, literature review.

## Funding

No funding was received for this manuscript.

## Disclosure

All authors read and approved the final manuscript.

## Ethics Statement

This report describes the clinical management of a single patient. According to the policy of Hopitaly Vaovao Mahafaly (Mandritsara, Madagascar), formal ethics committee approval is not required for anonymised case descriptions. Written informed consent for participation and publication was obtained from the patient.

## Consent

Written informed consent was obtained from the patient for publication of this anonymised case and the accompanying clinical details.

## Conflicts of Interest

The authors declare no conflicts of interest.

## Author Biographies

Lesley Notghi is a UK‐trained clinician who has served as a volunteer doctor at Hopitaly Vaovao Mahafaly, Madagascar, since 2017. Louise Little is a long‐term volunteer nurse leading the High‐Dependency Unit at the same institution. Joel Chin is an anaesthetist at Cambridge University Hospitals NHS Foundation Trust, United Kingdom, and previously worked at Hopitaly Vaovao Mahafaly and helped to set up the HDU.

## Data Availability

The data that support the findings of this study are available from the corresponding author upon reasonable request.
